# Weakened Contractile Performance and Mitochondrial Respiratory Complex Activity in Skeletal Muscle Improve during Interbout Arousal in Hibernating Daurian Ground Squirrel, *Spermophilus dauricus*

**DOI:** 10.3390/ijms242115785

**Published:** 2023-10-30

**Authors:** Huiping Wang, Yuxi Guo, Wenjing Yan, Liqi Cao, Xiaozhuo Bai, Jing Zhao, Kai Dang, Yunfang Gao

**Affiliations:** 1 Shaanxi Key Laboratory for Animal Conservation, College of Life Sciences, Northwest University, Xi’an 710069, China; wanghp@nwu.edu.cn (H.W.); 202233103@stumail.nwu.edu.cn (Y.G.); 202221258@stumail.nwu.edu.cn (W.Y.); 202233066@stumail.nwu.edu.cn (L.C.); 202332791@stumail.nwu.edu.cn (X.B.); 202332648@stumail.nwu.edu.cn (J.Z.); 2 Key Laboratory of Resource Biology and Biotechnology in Western China, College of Life Sciences, Northwest University, Xi’an 710069, China; 3 Lab for Bone Metabolism, Xi’an Key Laboratory of Special Medicine and Health Engineering, School of Life Sciences, Northwestern Polytechnical University, Xi’an 710072, China

**Keywords:** contractile properties, mitochondrial respiratory chain complex, skeletal muscle, hibernation, interbout arousal, Daurian ground squirrels

## Abstract

Mammalian hibernation is composed of multiple episodes of torpor bout, separated by phases of interbout arousal. During torpor, the skeletal muscles of mammals are undoubtedly inactive, but it has been proven to mitigate disuse atrophy. While interbout arousal has been implicated in the prevention of muscle atrophy, the underlying mechanisms sustaining muscle contraction remain to be explored. In the present study, Daurian ground squirrels (*Spermophilus dauricus*) were divided into four groups: pre-hibernation (PRE), torpor (TOR), interbout arousal (IBA), and post-hibernation (POST). The contractile performance of slow-twitch soleus muscle (SOL) and fast-twitch extensor digitorum longus muscle (EDL) was detected both in situ and in vitro. Concurrently, mitochondrial respiratory chain complex activity in these muscles was quantified. Our findings revealed that in situ contractile properties of both muscles, including force, power output, time duration, and force development/relaxation rates of twitch contraction, and force and power output of tetanic contraction declined in the TOR group compared to the PRE group, but improved in the IBA and POST groups. Fatigue resistance of muscles, determined by the power output of repetitive tetanic contractions in situ, decreased in the TOR group but recovered in the IBA and POST groups. In vitro studies demonstrated that tetanic contraction power output in isolated muscles increased with muscle temperature in both TOR and IBA groups. However, at the same temperature, power output was consistently lower in the TOR group compared to the IBA group. Moreover, the activity of the mitochondrial respiratory chain complex, especially Complexes I and II, decreased in the TOR group but showed recovery in the IBA and POST groups. These findings suggest that both the contractile performance and fatigue resistance of mammalian skeletal muscle are compromised during torpor but can be improved during interbout arousal and post-hibernation. The rebound in body temperature and rise in mitochondrial respiratory chain complex activity in skeletal muscle are involved in enhancing contractile performance and fatigue resistance. This study suggests that interbout arousal functions as a vital temporal interval during which skeletal muscles can transition from the inactivity induced by torpor to a state of restored contractile functionality. Thus, interbout arousal serves as a behavioral safeguard against disuse-induced damage to skeletal muscles during hibernation.

## 1. Introduction

Hibernation is an adaptive behavioral strategy employed by many mammalian species to endure harsh environmental conditions. During this prolonged period, which may last several months, mammals keep themselves in reduced respiration frequency, low body temperature, diminished metabolic rate, and rare body movement, to minimize energy and substrate expenditure [1,2,3]. Lacking of intake and load for a long time, the contractile function of skeletal muscle in many hibernating species deteriorates during hibernation. For example, late-hibernating black bears (*Ursus americanus*) exhibit a 29% reduction in twitch force of the tibialis anterior muscle compared to those in early hibernating [4]. Similarly, white-tailed prairie dogs (*Cynomys leucurus*) show a 25% and 16% reduction in force in the extensor digitorum longus and soleus muscles after winter hibernation, respectively, while black-tailed prairie dogs (*Cynomys ludovicianus*) exhibit corresponding declines of 22% and 10% [5]. Remarkably, however, hibernating mammals do not experience skeletal muscle disuse atrophy to the same extent observed in non-hibernating mammals under disuse conditions [6,7]. The potential mechanisms enabling hibernating mammals to resist muscle atrophy require further exploration.

Mammalian hibernation consists of several non-periodic torpor bouts, interspersed with periods of interbout arousal. During the arousal period, the torpid individual becomes active and regains a normothermic body temperature through thermogenesis, usually lasting less than 24 h. Studies have suggested that during arousal episodes, torpid animals can restore body water and electrolyte balance [8,9], replenish enzymes required for cardiac function at low body temperature [10,11], and initiate immune responses [12]. These observations imply that arousal may play an important role in promoting survival and maintaining physiological health during hibernation. However, the specific effects of arousal on skeletal muscle contractile performance, as well as its role in counteracting muscle atrophy during hibernation, have yet to be elucidated. A previous study on bats (*Murina leucogaster*) reported that the maximum tetanic tension and rate of tetanic tension development in the biceps brachii muscle during interbout arousal were comparable to those in summer-active and winter-torpor, indicating that arousal may help preserve muscle contractile properties and thus overcome muscle atrophy despite prolonged disuse during dormancy [13].

The primary aim of this present study is to explore the changes in the contractile properties of skeletal muscle in hibernating mammals during winter hibernation and assess the effects of interbout arousal on muscle maintenance. The former studies on skeletal muscle contraction in hibernators have primarily relied on in vitro techniques, isolating and maintaining muscles in buffer solution at a fixed temperature, such as 37 °C [5,14] or 25 °C [13]. However, subjecting the muscles of torpid and non-torpid individuals to the same temperature fails to take into account the differential effects of body temperature on muscle contractile performance across these physiological states. The contractile performance of mammalian skeletal muscles is temperature-sensitive [15,16], and this sensitivity also extends to the isometric contractile properties of skeletal muscles in hibernating torpid mammals [17]. Therefore, we employed an in situ method in the present study to measure muscle contraction, meticulously preserving both nerve connections and blood supply to the muscle. This methodology enabled the muscle to function in an environment approximately consistent with its internal physiological state, thereby offering a more accurate depiction of skeletal muscle contractile performance across different body temperatures. We also assessed the contractile performance of skeletal muscles in individuals under different hibernation states and temperatures via an in vitro method to clarify the role of body temperature on muscle contraction. In addition, we evaluated the activity of the mitochondrial respiratory chain complex within muscles to explore the changes in mitochondrial functionality during hibernation.

## 2. Results

### 2.1. Body and Muscle Mass

**Figure 1:**
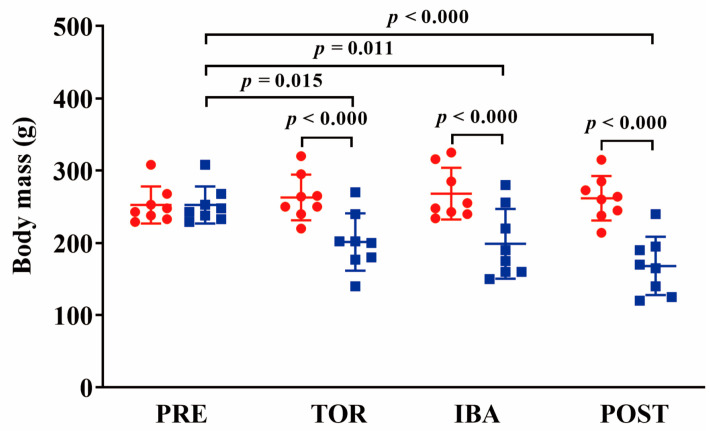
Changes in body mass among groups. Red: distribution of body mass at grouping time; Blue: distribution of body mass at sampling time. *n* = 8. Paired-samples *t*-test was used to compare body mass in the same group.

**Figure 2:**
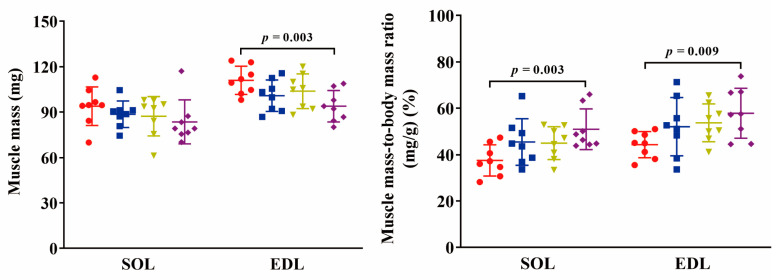
Changes in muscle mass and muscle mass-to-body mass ratio among groups. Red: PRE group; Blue: TOR group; Green: IBA group; Purple: POST group. *n* = 8. One-way ANOVA and Fisher’s LSD post hoc comparison were used to compare differences among groups.

Individual body mass distributions among the four groups were similar at the initial group division. At sampling, the mean body mass in torpor (TOR), interbout arousal (IBA), and post-hibernation (POST) groups decreased by 24% (*p* < 0.05), 27% (*p* < 0.05), and 37% (*p* < 0.001), respectively, compared to that at initial grouping (Figure 1). SOL muscle mass showed no significant differences among the different groups (*p* > 0.05). The muscle mass-to-body mass ratio was significantly increased in the post-hibernation (POST) group (36%, *p* < 0.01) compared to the pre-hibernation (PRE) group. Extensor digitorum longus (EDL) muscle mass was significantly decreased in the POST group (15%, *p* < 0.01) compared to the PRE group. The muscle mass-to-body mass ratio was significantly increased in the POST group (31%, *p* < 0.01) compared to the PRE group (Figure 2).

### 2.2. Twitch Contraction In Situ

**Figure 3:**
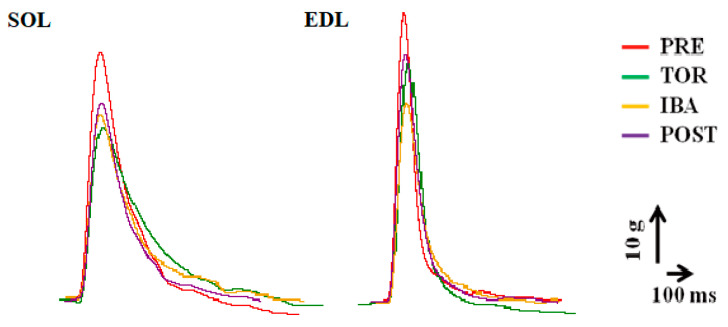
Twitch contraction curves among groups.

**Figure 4:**
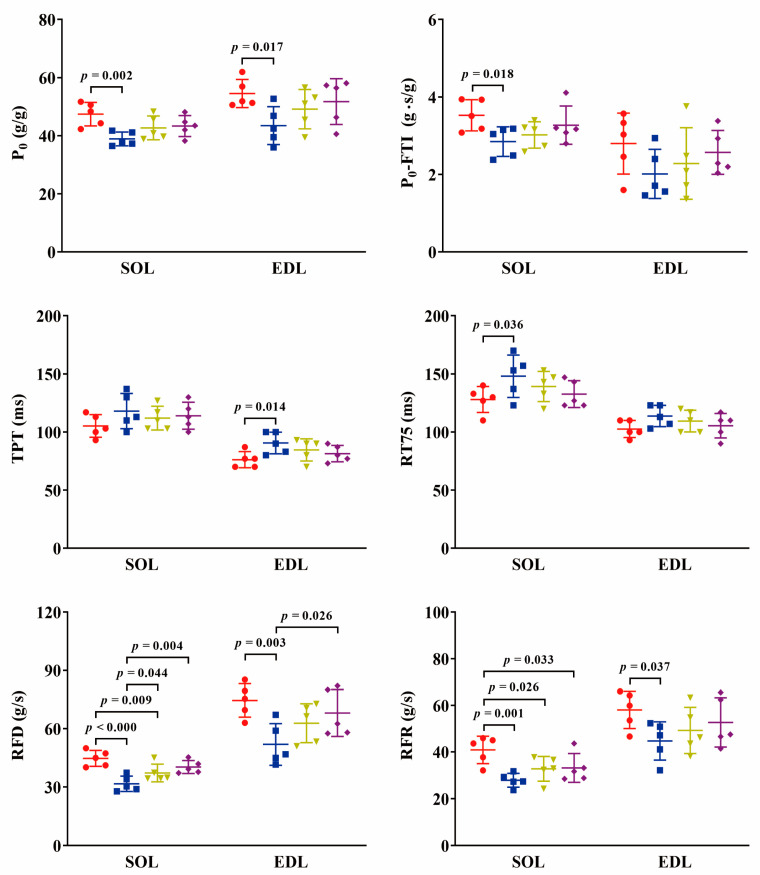
Changes in twitch contraction properties among groups. Red: PRE group; Blue: TOR group; Green: IBA group; Purple: POST group. P_0_: maximum twitch force; P_0_-FTI: force-time integral of P_0_; TPT: twitch time from onset to peak; RT75: relaxation time from peak to 75% force; RFD: rate of twitch force development; RFR: rate of twitch force relaxation. *n* = 6. One-way ANOVA and Fisher’s LSD post hoc comparison were used to compare differences among groups.

The twitch contraction curve and associated properties are illustrated in Figure 3 and Figure 4. In the SOL muscle, the maximum twitch force (P_0_) exhibited a significant reduction (18%, *p* < 0.01) in the TOR group compared to the PRE group, while showed a non-significant increase in the IBA and POST groups. The twitch time from onset to peak (TPT) and relaxation time from peak to 75% force (RT75) were prolonged in the TOR group relative to the PRE group (*p* > 0.05 and *p* < 0.05) but were shorter in the IBA and POST groups compared to the TOR group. The rate of twitch force development (RFD) was obviously slower in the TOR group than in the PRE group (*p* < 0.001). Although the IBA and POST groups displayed faster RFD values compared to the TOR group (*p* < 0.05 and *p* < 0.01, respectively), they still remained slower than the PRE group (*p* < 0.01 and *p* > 0.05, respectively). Similarly, the rate of twitch force relaxation (RFR) was significantly slower in the TOR group than in the PRE group (*p* < 0.01). While the IBA and POST groups displayed increased RFR speeds compared to the TOR group, they were 20% (*p* < 0.05) and 19% (*p* < 0.05) slower, respectively, than the PRE group. Furthermore, the force-time integral of P_0_ (P_0_-FTI) decreased by 19% (*p* < 0.05) in the TOR group compared to the PRE group but showed improvement in the IBA and POST groups, approaching levels observed in the PRE group.

In the EDL muscle, P_0_ exhibited a significant reduction (20%, *p* < 0.05) in the TOR group compared to the PRE group and then a non-significant enhancement in the IBA and POST groups, approaching levels observed in the PRE group. Both TPT and RT75 were prolonged in the TOR group compared to the PRE group (*p* < 0.05 and *p* > 0.05, respectively) but were shortened in the IBA and POST groups compared to the TOR group. The RFD and RFR declined by 30% (*p* < 0.01) and 23% (*p* < 0.05) in the TOR group compared to the PRE group. Conversely, the RFD and RFR increased in the IBA and POST groups to comparable levels observed in the PRE group. Similarly, the P_0_-FTI also decreased in the TOR group but improved in the IBA and POST groups.

### 2.3. Tetanic Contraction In Situ

**Figure 5:**
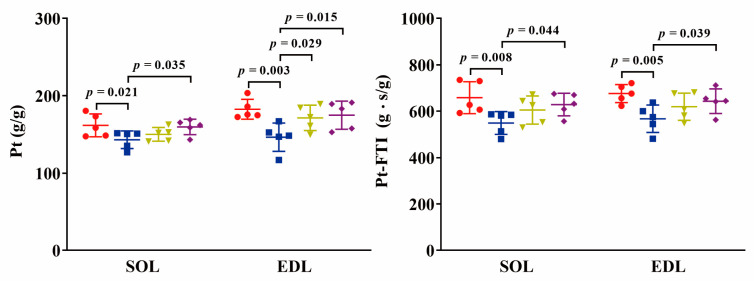
Changes in tetanic contraction properties among groups. Red: PRE group; Blue: TOR group; Green: IBA group; Purple: POST group. *n* = 6. One-way ANOVA and Fisher’s LSD post hoc comparison were used to compare differences among groups.

**Figure 6:**
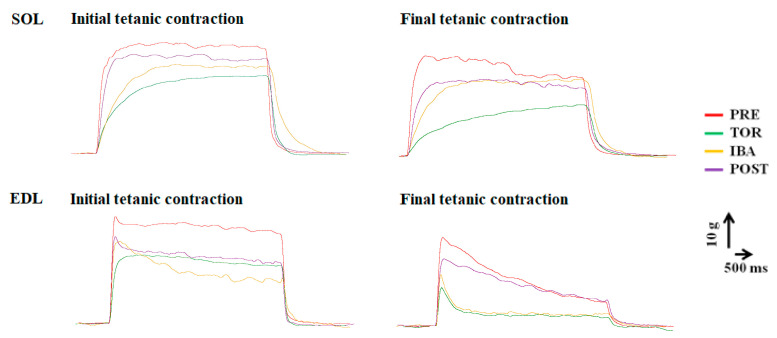
Initial and final tetanic contraction curves during repetitive tetanic contractions for fatigability test among groups. The initial tetanic contraction curve also serves as the tetanic contraction curve.

The associated properties in tetanic contraction are shown in Figure 5. The tetanic contraction curves are shown in Figure 6 (see the initial tetanic contraction curves). In both SOL and EDL muscles, the maximum tetanic force (Pt) displayed a significant decline in the TOR group (12%, *p* < 0.05 and 20%, *p* < 0.01, respectively) compared to the PRE group. Conversely, the Pt increased in the IBA and POST groups, reaching levels similar to that of the PRE group. The force-time integral of Pt (Pt-FTI) of SOL and EDL muscles also exhibited a significant decline in the TOR group (17%, *p* < 0.01 and 16%, *p* < 0.01, respectively) compared to the PRE group. However, the Pt-FTI increased in the IBA and POST groups, reaching levels similar to that of the PRE group.

### 2.4. Fatigue Resistance In Situ

**Figure 7:**
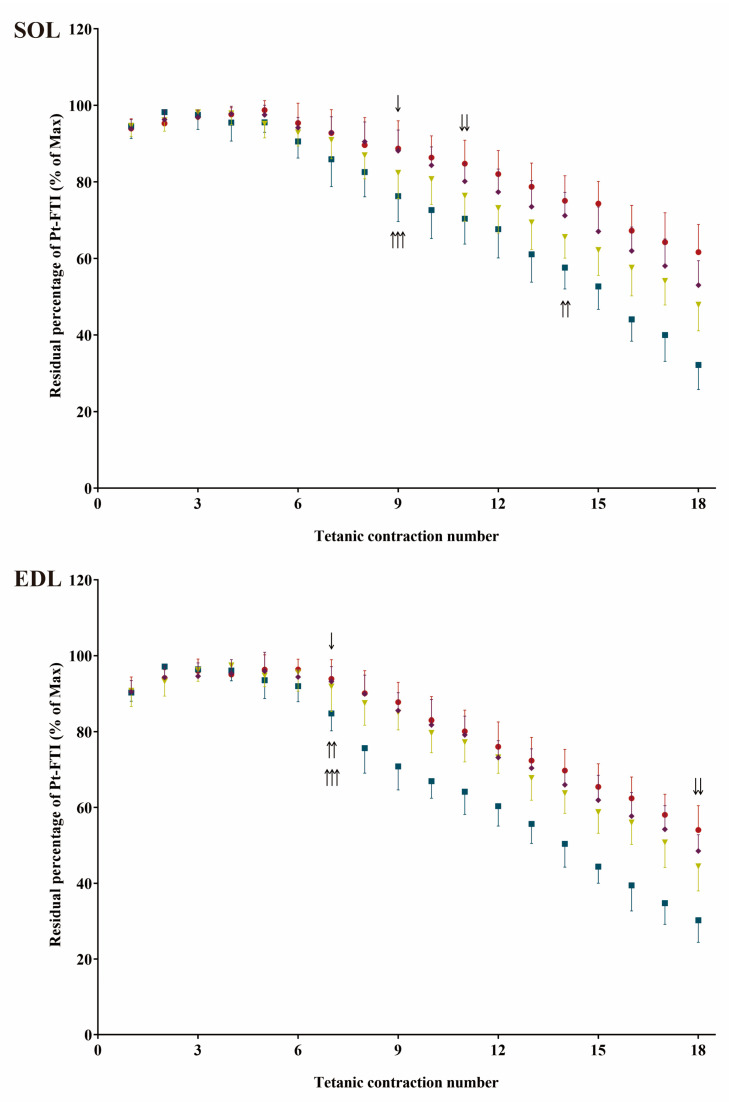
Changes in Pt-FTI during repetitive tetanic contractions for fatigability test among groups. Pt-FTI at each repetitive tetanic contraction was standardized as a percentage of maximum Pt-FTI during repetitive tetanic contractions. Red: PRE group; Blue: TOR group; Green: IBA group; Purple: POST group. *n* = 5. One-way ANOVA and Fisher’s LSD post hoc comparison were used to compare differences among groups at the same repetitive contraction number. Differences between TOR and IBA groups with the PRE group first reaching significance (*p* < 0.05) were indicated by “↓” and ”↓↓”, respectively. Differences between IBA and POST groups with the TOR group first reaching significance (*p* < 0.05) were indicated by “↑↑” and “↑↑↑”, respectively.

The initial and final tetanic contraction curves during repetitive tetanic contractions for the fatigability test are shown in Figure 6. The changes in Pt-FTI during the fatigability test are shown in Figure 7. (The changes in Pt during the fatigability test are shown in Supplementary Figure S1.) The results showed that, during repetitive tetanic contractions, the Pt-FTI in both muscles declined at markedly rapid rates in the TOR group, as opposed to the more moderate rates observed in the IBA and POST groups. The Pt-FTI was the weakest in the TOR group among all groups (ANOVA main effect, *F* = 73.40, *p* < 0.001 in SOL and *F* = 104.82, *p* < 0.001 in EDL). Starting from the 9th contraction in SOL and the 7th contraction in EDL, the residual percentages of Pt-FTI were significantly lower in the TOR group than in the PRE group. Starting from the 11th contraction in SOL and the 18th contraction in EDL, the residual percentages of Pt-FTI were significantly lower in the IBA group than in the PRE group. The residual percentages of Pt-FTI at each contraction in the POST group showed no significant differences compared to the PRE group. Starting from the 14th contraction in SOL and the 7th contraction in EDL, the residual percentages of Pt-FTI were significantly higher in the IBA group than in the TOR group. Starting from the 9th contraction in SOL and the 7th contraction in EDL, the residual percentages of Pt-FTI were significantly higher in the POST group compared to the TOR group (One-way ANOVA and Fisher’s LSD post hoc comparison, *p* < 0.05 in each case). Moreover, significant differences were observed in the shape of Pt-FTI fatigue curves (ANOVA interaction term, *F* = 2.80, *p* < 0.001 in SOL; *F* = 3.51, *p* < 0.001 in EDL). The results indicated that the TOR group experienced the most rapid muscle fatigue relative to the other groups.

### 2.5. Twitch and Tetanic Contraction In Vitro

**Figure 8:**
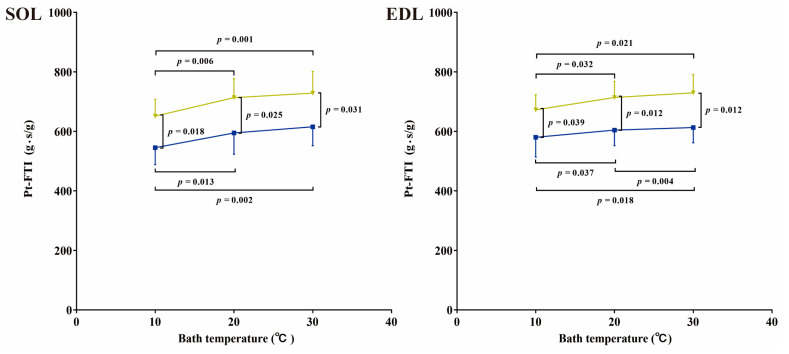
Changes in Pt-FTI at various temperature points in vitro in TOR and IBA groups. Blue: TOR group; Green: IBA group. *n* = 5. Paired-samples *t*-test was used to compare differences between various temperature points within the same group, and an independent-sample *t*-test was used to compare differences between TOR and IBA groups at the same temperature point.

The changes in Pt-FTI in vitro at various temperature points are depicted in Figure 8. (The changes in P_0_, P_0_-FTI, and Pt in vitro at various temperature points are depicted in Supplementary Figures S2, S3, and S4, respectively.) In both SOL and EDL muscles, Pt-FTI increased as the bath temperature increased in the TOR group, showing significantly lower values at 10 °C than at 20 °C and 30 °C (*p* < 0.05). Meanwhile, Pt-FTI decreased as the bath temperature decreased in the IBA group, showing significantly lower values at 10 °C than at 20 °C and 30 °C (*p* < 0.05). In addition, Pt-FTI levels were significantly higher in the IBA group than those in the TOR group at the same temperature (*p* < 0.05).

### 2.6. Mitochondrial Respiratory Chain Complex Activity

**Figure 9:**
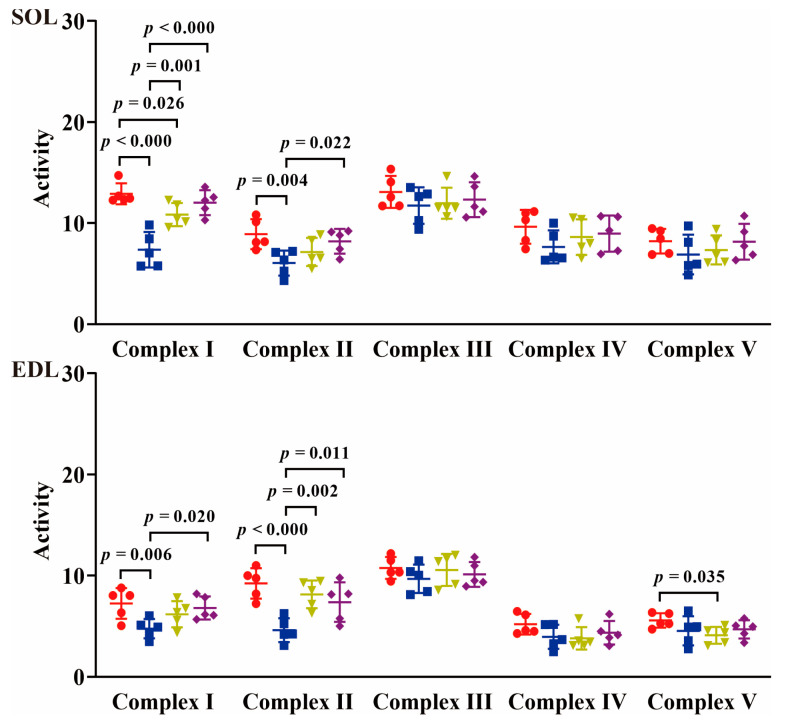
Changes in mitochondrial respiratory chain complex activity among groups. Red: PRE group; Blue: TOR group; Green: IBA group; Purple: POST group. Units: Complex I, μmol NADH/min/mg prot; Complex II, μmol DCPIP/min/mg prot; Complex III, μmol CoQH_2_/min/mg prot; Complex IV, μmol cytochrome c/min/mg prot; Complex V, μmol NADH/min/mg prot. *n* = 5. One-way ANOVA and Fisher’s LSD post hoc comparison were used to compare differences among groups.

The changes in mitochondrial respiratory chain complex activity are depicted in Figure 9. In SOL muscle, the activity levels of all complexes declined in the TOR group. Specifically, the activities of Complexes I and II exhibited significant reductions of 43% and 32% (*p* < 0.001 and *p* < 0.01), respectively, compared to those in the PRE group. Conversely, the activities of Complexes I and II increased by 47% and 18% (*p* < 0.01 and *p* > 0.05) in the IBA group and by 63% and 36% (*p* < 0.01 and *p* < 0.05) in the POST group, respectively, compared to those in the TOR group. These levels approximated PRE group levels, except that Complex I activity was significantly lower (16%, *p* < 0.05) in the IBA group. In addition, no significant changes were observed in the activities of Complexes III, IV, and V among groups. In the EDL muscle, the activity levels of all complexes declined in the TOR group. Specifically, the activities of Complexes I and II exhibited significant reductions of 34% and 50% (*p* < 0.01 and *p* < 0.001), respectively, compared to those in the PRE group. Conversely, the activities of Complexes I and II increased by 30% and 77% (*p* > 0.05 and *p* < 0.01) in the IBA group and by 43% and 60% (*p* < 0.05 and *p* < 0.05) in the POST group, respectively, compared to those in the TOR group, thus approaching PRE group levels. In addition, no significant differences were observed in the activities of Complexes III, IV, and V among groups, except that the activity of Complex V was significantly lower (*p* < 0.05) in the IBA group than in the PRE group.

## 3. Discussion

In the present study, we examined the contractile performance of skeletal muscles in Daurian ground squirrels across various hibernation states to explore changes in contractile function during hibernation and the potential underlying mechanisms. Given that skeletal muscles can be classified into fast- and slow-twitch types based on their contractile performance, we selected soleus (SOL), a typical slow-twitch muscle, and extensor digitorum longus (EDL), a typical fast-twitch muscle, for the present study. Our findings revealed that the changes in contractile properties of both SOL and EDL muscles during hibernation were similar and did not exhibit significant differences based on their distinct twitch types. Consequently, we discuss the results for both muscle types collectively.

### 3.1. Body and Skeletal Muscle Mass Are Lost in Daurian Ground Squirrels during Hibernation

Ground squirrels that experienced several bouts of torpor exhibited a loss in body mass, and the loss was more pronounced in individuals that had undergone more torpor bouts in hibernation season. Meanwhile, such loss was also observed in skeletal muscle mass in these individuals. These findings are consistent with previous studies, including our own research [18,19,20]. These reductions can be attributed to continuous energy expenditure without intake during hibernation. As the loss of body mass occurred more rapidly and severely than that of muscle mass, there was a resultant increase in the muscle mass-to-body mass ratio as hibernation progressed. Given the observed muscle mass loss during hibernation, we suggest normalizing muscle contractile properties to muscle mass for a more accurate quantification of contraction changes.

### 3.2. Muscle Contractile Performance Is Poor in Torpor but Improves during Interbout Arousal and Post-Hibernation

The in situ detection in the present study revealed a decline in both twitch and tetanic contractile properties of skeletal muscles during hibernation torpor. Such impaired contractile performance has also been documented in other hibernating species, such as torpid white-tailed prairie dogs, black-tailed prairie dogs, and black bears [4,5]. However, our study further demonstrated that the depressed contractile performance exhibited a marked improvement during interbout arousal and post-hibernation stages. Such functional recovery confers survival advantages and facilitates survival behaviors, such as prey capture and predator avoidance, during interbout arousal and post-hibernation. Thus, we hypothesize that the recovery of skeletal muscle contractile function during interbout arousal may serve as a self-protective adaptation mechanism to facilitate animals’ survival during hibernation.

Regarding the underlying causes of the observed variations in skeletal muscle contractile performance across different hibernation states, several factors merit consideration. The one is the muscle fiber-type transition which happens during hibernation. The shift from fast-twitch (type II) to slow-twitch (type I) fibers had been found in both slow (diaphragm, soleus) and fast (plantaris, gastronomies, and extensor digitorum longus) muscles in torpid ground squirrels [18,19,20,21,22]. Additionally, mammalian hibernators have been reported to show an average 25% increase in the ratio of type I fibers during hibernation compared to the summer active period [7]. Different types of muscle fiber show differences in contractile properties. For example, the rate of twitch force development (RFD) varies among different myosin heavy chain isoforms, following the sequence types IIA/X, IIA, I/IIA, and I, from highest to lowest [23]. In the present study, the extended duration of twitch and relaxation times, along with the reduced rates of force development and relaxation in twitch contraction in hibernating torpid individuals, may be indicative of such muscle fiber-type transition.

However, the muscle fiber-type transition does not fully account for the timely recovery of contractile performance observed during interbout arousal. Muscle contraction, resulting from the interactions and movements between protein molecules, is sensitive to temperature. The body temperature of a hibernating torpid squirrel is severely lower than that of a squirrel in other hibernation states. In this hibernation state, the squirrel’s contractile performance is also extremely poor. When the individual is arousing or emerging from hibernation, its body temperature rises, and at the same time, its contractile performance also recovers. In this study, we further observed the performance of muscle contraction under different muscle temperatures in vitro. The results reveal that all contractile properties improved effectively as the muscle temperature increased. Thus, body temperature variations at different hibernation states may be a contributing factor to the observed changes in skeletal muscle contractile function.

In addition, our results also indicated variations in skeletal muscle contractile performances between torpid and interbout-aroused squirrels, even under the same temperature conditions in vitro. This suggests the presence of certain intrinsic factors within the muscle that contribute to variations in skeletal muscle contraction. The role of energy generation as a contributing factor will be further explored in subsequent sections.

### 3.3. Skeletal Muscle Is More Susceptible to Fatigue during Torpor but Recovers during Interbout Arousal and Post-Hibernation

In the present study, torpid ground squirrels exhibited a severely rapid decline in both force and power output in skeletal muscle during repetitive tetanic contractions, pointing to extremely low resistance to muscle fatigue. During interbout arousal or post-hibernation, the individuals displayed a marked improvement in fatigue resistance. Given body temperature variations at different hibernation states, it is necessary to consider the effects of body temperature when exploring the impact of hibernation on skeletal muscle fatigue resistance. Previous research has reported that wild black bears (*Ursus americanus*) can maintain skeletal muscle fatigue resistance during hibernation [4]. However, it should be noted that these torpid bears experience only a modest reduction in body temperature in contrast to small hibernation mammals, whose body temperature presents a remarkable decrease during torpor. James et al. demonstrated that when skeletal muscles isolated from both torpid and summer-active 13-lined ground squirrels (*Ictidomys tridecemlineatus*) were maintained at the same temperature (36.5 ± 0.5 °C), the fatigue resistance in torpid squirrels remained substantially lower than that in their summer-active counterparts [14]. In this study, we also found that, although the power output of skeletal muscles from torpid squirrels increased as muscle temperature increased in vitro, it was still lower than that in the interbout-aroused squirrels at the same temperature. These collective findings suggest that alterations in skeletal muscle fatigue resistance throughout hibernation in small mammals cannot be solely attributed to body temperature fluctuations but may also involve intrinsic changes within the muscle tissue. To substantiate this hypothesis, we evaluate the energy supply in muscles by measuring the activity of the mitochondrial respiratory chain complex across different hibernation states.

### 3.4. Mitochondrial Respiratory Chain Complex Activity Declines during Torpor but Recovers during Interbout Arousal and Post-Hibernation

The mitochondrial respiratory chain is composed of a group of enzyme complexes that facilitate the transfer of electrons generated during oxidative phosphorylation. The electron transfer is accompanied by adenosine triphosphate (ATP) synthesis and heat release. As mammals enter into hibernation torpor, mitochondrial respiration drops to extremely low levels [24,25,26]. Previous studies have pointed out that the activity of the mitochondrial respiratory chain complex was depressed in hibernating animals [27,28]. Our findings also revealed a decline in the activity of all complexes (Complexes I–V) in the skeletal muscles of torpid ground squirrels. The reduction in complex activity could diminish both the efficiency and overall output of mitochondrial energy production, thereby negatively affecting skeletal muscle contraction, which is highly reliant on intracellular energy. The former study suggested that low skeletal muscle fatigue resistance in torpid 13-lined ground squirrels was related to a decrease in mitochondrial oxidative capacity [14]. Therefore, we infer that the reduced activity in the mitochondrial respiratory complex may be a contributing factor to the poor contractile performance and low fatigue resistance observed in the skeletal muscles of torpid squirrels. We also found that the activity of all complexes increased during interbout arousal or emergence from hibernation, suggesting that this increase may play a role in the recovery of contractile performance and fatigue resistance.

Moreover, our results also indicated that the activity fluctuations were more pronounced in Complexes I and II than in other complexes. Complex I (NADH–ubiquinone oxidoreductase) and Complex II (succinate–ubiquinone oxidoreductase) are crucial enzymes that facilitate the entry of NADH and FADH_2_ into the electron transport chain and the transfer of electrons to Coenzyme Q (CoQ), respectively, thereby serving as the gateway for oxidative phosphorylation. Previous research on golden-mantled ground squirrels (*Callospermophilus lateralis*) had pointed out that fatty acids exert considerable suppressive effects on the oxidative phosphorylation capacity of Complex (I + II) in the heart, muscle, and liver mitochondria [29]. The fatty acids as the fuel have been reported in mammalian hibernation [30,31,32]. We speculate that modulating the activity of Complexes I and II through fatty acids may be a direct and effective mechanism for regulating skeletal muscle mitochondrial respiratory efficiency during mammalian hibernation.

### 3.5. Interbout Arousal May Serve as a Protective Mechanism to Prevent Skeletal Muscle Disuse Atrophy during Mammalian Hibernation

Harlow et al. proposed that maintaining fiber-type integrity and muscle strength in torpid black bears throughout the winter denning period requires a certain amount of muscle activity, including both voluntary contractions and involuntary shivering [33]. Similarly, Lee et al. suggested that arousal accompanied by intense muscle shivering and heat stress, as well as up-regulation of heat shock proteins, contributes to the preservation of skeletal muscle properties during prolonged disuse in bats [13]. Based on the current findings, we posit that interbout arousal may serve as an important time window for skeletal muscle to interrupt the inactivity imposed by torpor and recover the contractile capacity. Furthermore, interbout arousal may function as a self-protective behavior in mammalian hibernation. During interbout arousal, the rebound in body temperature and restoration of mitochondrial energy production in skeletal muscle improve muscle contractility, thereby enabling hibernating mammals to counteract muscle atrophy arising from prolonged disuse during hibernation torpor.

## 4. Materials and Methods

### 4.1. Animals and Groups

Daurian ground squirrels (*Spermophilus dauricus*) of both sexes were trapped from the Weinan region in Shaanxi Province, China. The animals were housed in an environment with natural light and temperature and raised individually in conventional plastic cages with standard laboratory rat chow and water provided ad libitum. Body temperature (Tb) was measured based on thermal imaging twice daily using a visual thermometer (Fluke VT04 Visual IR Thermometer, Fluke, Everett, WA, USA) throughout the hibernation period.

In October, the squirrels were matched by body mass and assigned into four groups: pre-hibernation (PRE), torpor (TOR), interbout arousal (IBA), and post-hibernation (POST) groups. Individuals in the PRE group, characterized by normal Tb, were sampled at this time. The remaining squirrels were transferred into a dark, cold room (4–6 °C) for hibernation. In winter, samples were collected from individuals in the TOR group, which had undergone at least 60 d since the first torpor bout and maintained a Tb of ≤8 °C for a minimum of 5 d in the current torpor bout. Similarly, samples were collected from individuals in the IBA group, which had undergone at least 60 d since the first torpor bout and reached a Tb of ≥30 °C for no more than 12 h in the current interbout arousal. In spring, individuals in the POST group were sampled after maintaining a normal Tb for a minimum of 3 d from arousal. For the sampling procedure, animals were sufficiently anesthetized with urethane (1.5 g/kg body mass) via intraperitoneal injection. Soleus (SOL) and extensor digitorum longus (EDL), as slow-twitch and fast-twitch muscles, respectively, were immediately used for the next analyses. After surgical intervention, the animals were sacrificed by an overdose injection of urethane.

### 4.2. Isometric Contraction Measurement In Situ

The SOL muscle was surgically exposed and mechanically separated by removing as much as possible of the myofascial connections to surrounding muscles, while preserving the integrity of nerve connections and blood supply. The ankle tendon of the muscle was cut and connected to a force transducer coupled to an input interface (PowerLab 8sp, ADInstruments, Bella Vista, Australia) for trace acquisition and analysis. The knee joint was firmly immobilized and aligned horizontally with both the muscle and force transducer. Adjacent muscles were positioned such that they allowed the SOL in an unconstrained contraction. Two thin platinum electrodes were gently placed in contact with the upper surface of the SOL and controlled by an electronic stimulator (SEN-3301, Nihon Kohden, Japan) to deliver pulse wave stimulation. The optimum length of the muscle (L_0_) was increased in 0.1 mm increments controlled by a stepper regulator until maximum twitch force was attained. Krebs–Henseleit solution (in mM: 118 NaCl, 4.75 KCl, 1.18 MgSO_4_, 24.8 NaHCO_3_, 1.18 KH_2_PO_4_, 10 glucose, 2.54 CaCl_2_, pH7.4) was used to maintain muscle surface moisture. Muscle, maintained at the optimum length, was equilibrated for 20 min before subsequent stimulation protocols. Twitch contraction was elicited by a single-square wave stimulus with a pulse duration of 25 ms at 5 V. Tetanic contraction was elicited by a burst train stimulus with a pulse duration of 10 ms at 50 Hz and 5 V for 5 s. Muscle fatigability was determined through repetitive tetanic contractions, elicited by a cycle train stimulus with a pulse duration of 20 ms at 33 Hz and 5 V for 5 s, followed by a 10 s duty cycle lasting 3 min, resulting in a total of 18 tetanic contractions. The muscle was rested for 10 min between two stimulation protocols.

Twitch and tetanic contractions of the EDL muscle were also measured using the same methods as above. The stimulation protocols for EDL were identical to those used for SOL except for the burst train stimulus (10 ms at 50 Hz and 5 V for 5 s).

Contractions were recorded at a 100/s sampled frequency and analyzed by Chart v5.0 software (ADInstruments, Australia). Contractile properties, including twitch time from onset to peak (TPT), relaxation time from peak to 75% force (RT75), maximum twitch force (P_0_), and maximum tetanic force (Pt), were measured. The rate of twitch force development (RFD) and rate of twitch force relaxation (RFR) were calculated using P_0_/TPT and 75%·P_0_/RT75, respectively. Force-time integral (FTI) of twitch and tetanic contraction, i.e., the area under the P_0_ curve and Pt curve (P_0_-FTI and Pt-FTI), were used to represent muscle power output. Given the loss of muscle mass during hibernation, certain contractile properties were normalized by muscle mass. Muscle fatigue resistance was determined by changes in Pt and Pt-FTI during repetitive tetanic contractions. To minimize the influence of noise, contraction and relaxation processes were calculated as the transition time between 10% and 90% of peak force rather than 0% and 100%.

### 4.3. Isometric Contraction Measurement In Vitro

To detect the influence of muscle temperature on contractile properties, SOL and EDL muscle samples from TOR and IBA groups were immediately isolated after anesthesia to measure isometric contraction in vitro. The ankle tendon of the muscle was connected as described above, while the knee tendon was connected to a metal anchor. The muscle was soaked in a temperature-controlled bath containing Krebs–Henseleit solution. To mimic changes in muscle temperature during the torpor-arousal cycle, isometric contractions were recorded at gradually increasing bath temperatures (10 °C, 20 °C, and 30 °C) in the TOR group and gradually decreasing bath temperatures (30 °C, 20 °C, and 10 °C) in the IBA group. At each temperature point, P_0_, P_0_-FTI, Pt, and Pt-FTI were measured as above.

### 4.4. Mitochondrial Respiratory Chain Complex Activity Measurement

To measure the activity of the muscle mitochondrial respiratory chain complex, mitochondria were first isolated using a commercial kit (G006, Jiancheng Bioengineering Institute, Nanjing, China). A fresh muscle sample, weighing about 0.1 mg, was cut and homogenized in cold lysate buffer (1:10, *w*/*v*). The homogenate was filtered through a 200-mesh cell sieve. After centrifugation (800× *g*, 5 min, 4 °C), the supernatant was gently transferred onto the top of 1.0 mL of extract buffer. Following centrifugation (15,000× *g*, 10 min, 4 °C), the mitochondria were enriched at the bottom. The mitochondria were then re-suspended in 0.2 mL of rinse buffer and centrifuged again (15,000× *g*, 10 min, 4 °C). The mitochondria, enriched at the bottom, were re-suspended in 0.1 mL of storage buffer. The mitochondria were subsequently ultrasonically vibrated to fully expose the respiratory complex and achieve maximal activity. Protein content in the mitochondria was measured using a BCA protein assay kit (34580, Thermo Fisher Scientific, Waltham, MA, USA).

All assays for mitochondrial respiratory chain enzyme (Complexes I–V) activity were based on previous reports [34,35,36], with slight modifications, and were conducted using commercial kits (A089-1, A089-2, A089-3, A089-4, and A089-5, Jiancheng Bioengineering Institute, Nanjing, China) and a spectrophotometer. For Complex I (NADH–ubiquinone oxidoreductase), NADH served as the oxidized substrate and decylubiquinone as the electron acceptor. The activity rate was measured by monitoring the decrease in absorbance at 340 nm at 30 °C over 3 min, following the addition of decylubiquinone. To inhibit Complex I as a test of activity specificity, rotenone was added. The specific activity of Complex I was calculated as the difference between the total rate of NADH oxidation and the rate after rotenone addition. For Complex II (succinate–ubiquinone oxidoreductase), succinate functioned as the oxidized substrate, with decylubiquinone and dichlorophenol-indophenol (DCPIP) as the electron acceptors. The activity rate was determined by measuring the reduction in absorbance at 600 nm at 30 °C over 5 min after adding DCPIP. The activity of Complex II was expressed as the rate of DCPIP reduction. For Complex III (ubiquinol–cytochrome c oxidoreductase), reduced decylubiquinone was the oxidized substrate with cytochrome c as the electron acceptor. The activity rate was quantified by observing the increase in absorbance at 550 nm at 30 °C over 2 min, following the addition of decylubiqinone. To inhibit Complex III as a test of activity specificity, antimycin-A was added. The specific activity of Complex III was calculated as the difference between the total rate of decylubiqinone oxidation and the rate after antimycin-A addition. For Complex IV (cytochrome c oxidase), reduced cytochrome c was the oxidized substrate. The activity rate was assessed by measuring the reduction in absorbance at 550 nm at 25 °C over 1 min after adding reduced cytochrome c. The activity of Complex IV was expressed as the rate of cytochrome c oxidation. For Complex V (ATP synthase), NADH was used as the oxidized substrate, using a coupled enzyme assay with pyruvate kinase and lactate dehydrogenase. The activity rate was ascertained by measuring the reduction in absorbance at 340 nm at 30 °C over 5 min after adding NADH. To inhibit Complex V as a test of activity specificity, oligomycin was used. The specific activity of Complex V was calculated as the difference between the total rate of NADH oxidation and the rate after oligomycin addition.

### 4.5. Statistical Analysis

All data were presented as mean ± standard deviation (s.d.). Levene’s test demonstrated that variances were equal in all cases. Changes in individual body mass between grouping and sampling times were evaluated with paired-samples *t*-test. Two-factor analysis of variance (ANOVA) was used to analyze effects on fatigue resistance, with hibernation state and repetitive tetanic contraction number (essentially, time elapsed during repetitive tetanic contraction) used as the two factors. The interaction term in this analysis should indicate whether the shape in the residual Pt or Pt-FTI curve is different, hence indicating the difference in the pattern of fatigue among groups. One-way ANOVA and Fisher’s LSD post hoc comparison were used to determine whether Pt and Pt-FTI were significantly different among groups at each repetitive tetanic contraction during the fatigue run. In other cases, differences among groups were determined using one-way ANOVA and Fisher’s LSD post hoc comparison. All statistical analyses were performed using SPSS v17.0 at a significance level of *p* = 0.05. All figures were constructed using GraphPad Prism v8.0.

## 5. Conclusions

During hibernation, characterized by prolonged nutrient deprivation and inactivity, the Daurian ground squirrels exhibited a significant decline in body and skeletal muscle mass. Notably, the rate of body mass loss was greater than that of muscle mass, resulting in an increase in the muscle mass-to-body mass ratio during hibernation. Concurrently, the skeletal muscle of torpid squirrels exhibited poor contractile properties and fatigue resistance. Furthermore, there was a marked reduction in the activity of the mitochondrial respiratory chain complex, especially Complexes I and II. Upon interbout arousal, both the contractile properties and fatigue resistance of skeletal muscle were substantially restored. Moreover, the mitochondrial respiratory chain complex activity was increased, and an obvious rise appeared in the activity of Complexes I and II. Our findings suggest that modulating the activity of the mitochondrial respiratory chain complex, particularly which of Complexes I and II, serves as a precise and effective mechanism for regulating mitochondrial energy production during the torpor-arousal cycle in mammalian hibernation. The observed recovery in skeletal muscle contractile function and fatigue resistance during arousal could partly be attributed to the rebound in body temperature and partly to the increase in mitochondrial energy production within skeletal muscle. Interbout arousal likely functions as a self-protective mechanism in hibernating mammals to mitigate the risk of skeletal muscle atrophy due to prolonged disuse during torpor. Consequently, upon emerging from hibernation, the animals are better equipped to maintain normal muscle contractile function, thereby improving their chances of survival.

## Data Availability

The data presented in this study are available upon request from the corresponding authors.

## Supplementary Materials

The following supporting information can be downloaded at https://www.mdpi.com/article/10.3390/ijms242115785/s1.

## Abbreviations

CoQ	Coenzyme Q
EDL	Extensor digitorum longus
FADH_2_	Flavine adenine dinucleotide (reduced form)
FTI	Force-time integral
IBA	Interbout arousal group
L_0_	Optimum length of muscle
NADH	Nicotinamide adenine dinucleotide (reduced form)
P_0_	Maximum twitch force
POST	Post-hibernation group
PRE	Pre-hibernation group
Pt	Maximum tetanic force
RFD	Rate of twitch force development
RFR	Rate of twitch force relaxation
RT75	Relaxation time from peak to 75% force
SOL	Soleus
Tb	Body temperature
TOR	Hibernation torpor group
TPT	Twitch time from onset to peak
