# Supplementary material for: Weakened Contractile Performance and Mitochondrial Respiratory Complex Activity in Skeletal Muscle Improve during Interbout Arousal in Hibernating Daurian Ground Squirrel, Spermophilus dauricus
Source: Int J Mol Sci. 2023 Oct 30;24(21):15785. doi: 10.3390/ijms242115785 (PMC10650195; doi:10.3390/ijms242115785)
Supplement: Supplementary file 1 [file ijms-24-15785-s001.zip › ijms-2650215-supplementary.pdf]

**Supplementary Figure S1**

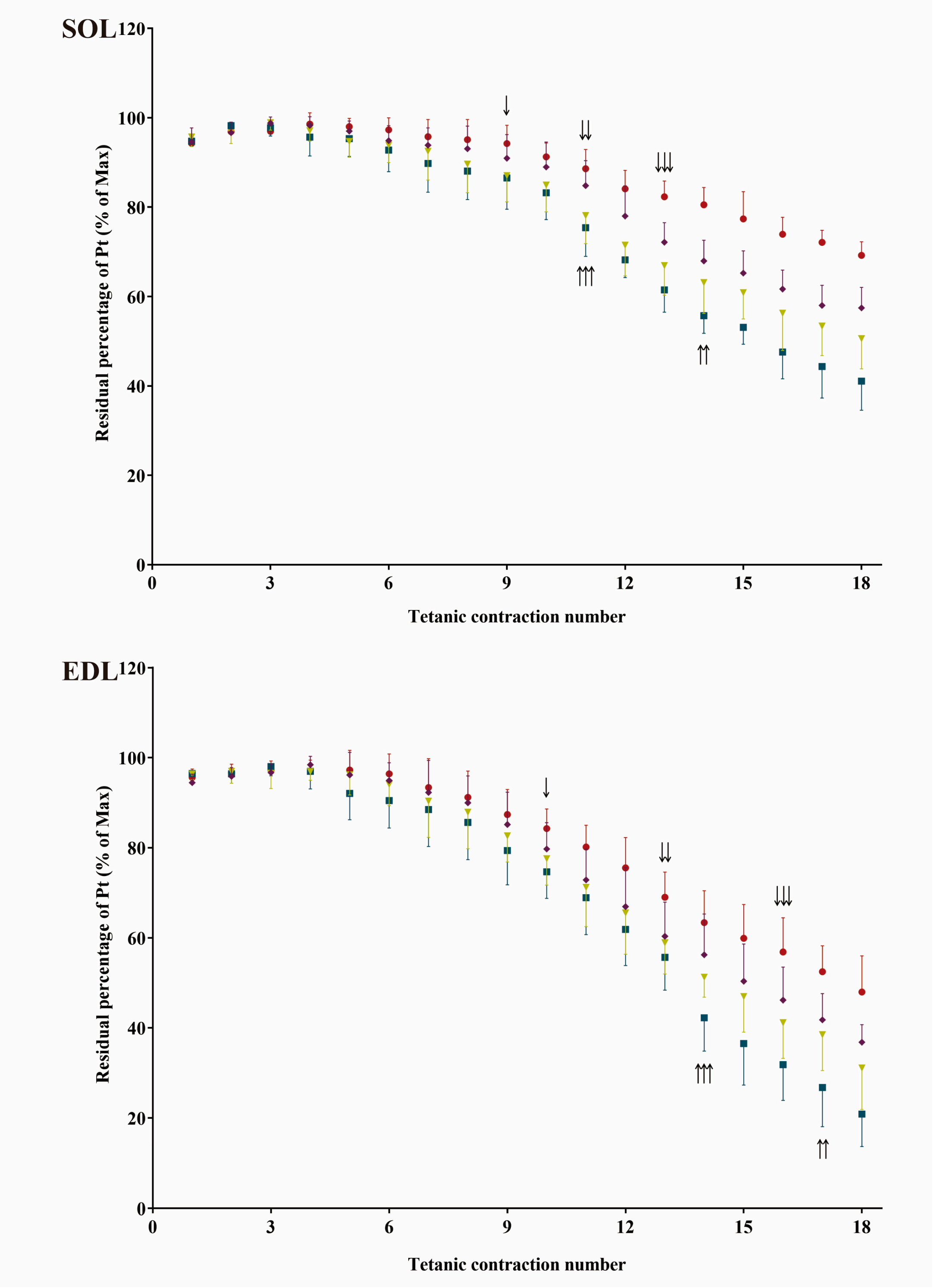


**Figure S1.** Changes in Pt during repetitive tetanic contractions for fatigability test among groups. Pt at each repetitive tetanic contraction was standardized as a percentage of the maximum Pt during repetitive tetanic contractions. Red: PRE group; Blue: TOR group; Green: IBA group; Purple: POST group. $n = 5$. One-way ANOVA and Fisher's LSD *post hoc* comparison were used to compare differences among groups at the same repetitive contraction number. Differences between TOR, IBA, and POST groups with PRE group first reaching significance ($p < 0.05$) were indicated by "↓", "↓↓", and "↓↓↓", respectively. Differences between IBA and POST groups with TOR group first reaching significance ($p < 0.05$) were indicated by "↑↑" and "↑↑↑", respectively.

## Supplementary Figure S2

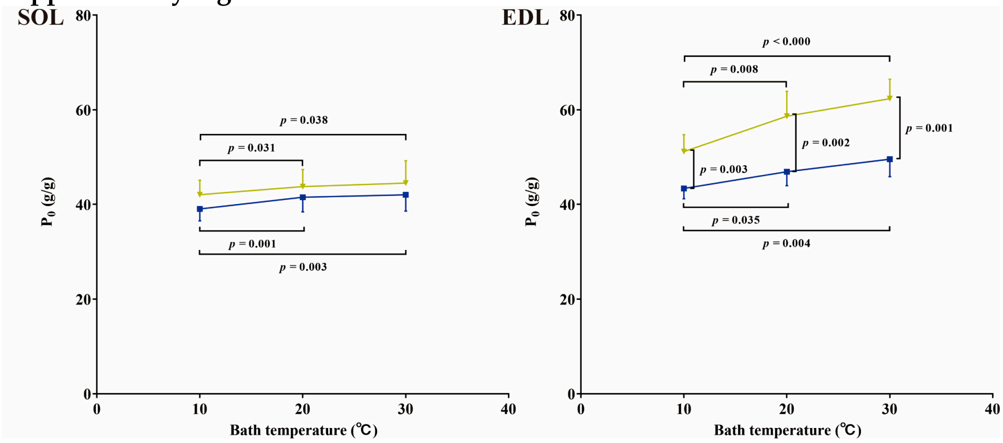


**Figure S2.** Changes in $P_0$ at various temperature points *in vitro* in TOR and IBA groups. Blue: TOR group; Green: IBA group. $n = 5$. Paired-samples $t$-test was used to compare differences between various temperature points within the same group, and independent-samples $t$-test was used to compare differences between TOR and IBA groups at the same temperature point.

## Supplementary Figure S3

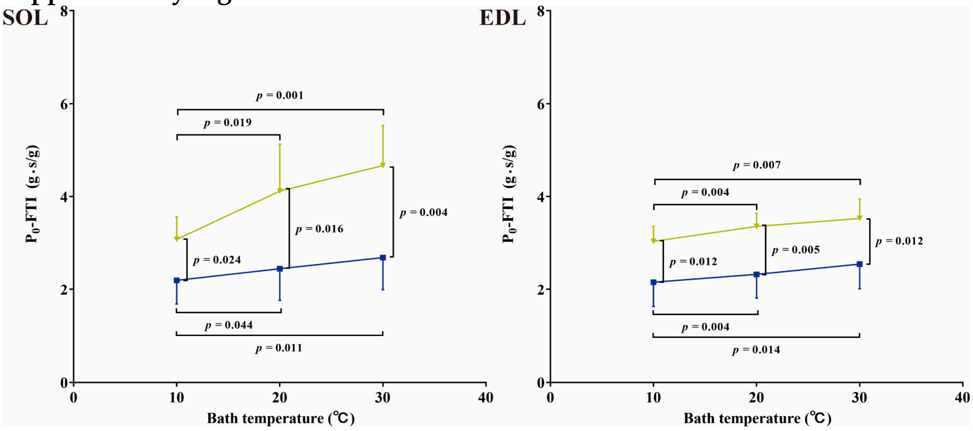


**Figure S3.** Changes in $P_0$-FTI at various temperature points *in vitro* in TOR and IBA groups. Blue: TOR group; Green: IBA group. $n = 5$. Paired-samples $t$-test was used to compare differences between various temperature points within the same group, and independent-samples $t$-test was used to compare differences between TOR and IBA groups at the same temperature point.

## Supplementary Figure S4

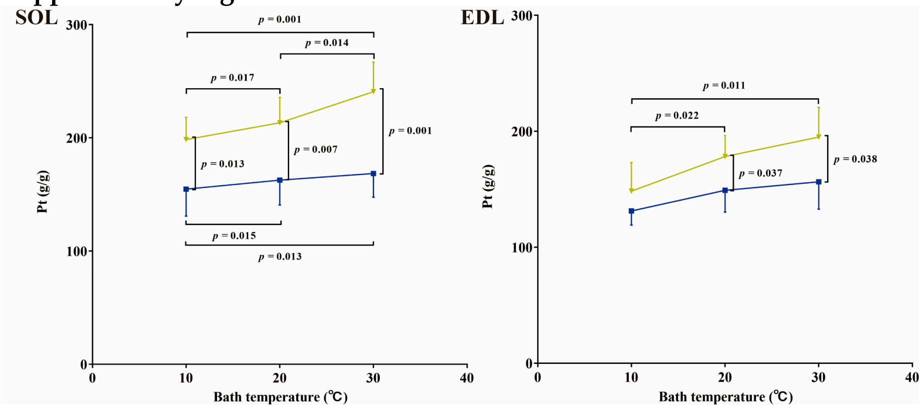


**Figure S4.** Changes in Pt at various temperature points *in vitro* in TOR and IBA groups. Blue: TOR group; Green: IBA group. $n = 5$. Paired-samples $t$-test was used to compare differences between various temperature points within the same group, and independent-samples $t$-test was used to compare differences between TOR and IBA groups at the same temperature point.
